# MRI follow-up after magnetic resonance-guided focused ultrasound for non-invasive thalamotomy: the neuroradiologist’s perspective

**DOI:** 10.1007/s00234-020-02433-9

**Published:** 2020-05-03

**Authors:** Vera C. Keil, Valeri Borger, Veronika Purrer, Simon F. Groetz, Lukas Scheef, Henning Boecker, Hans H. Schild, Christine Kindler, Angelika Schmitt, Laszlo Solymosi, Ullrich Wüllner, Claus C. Pieper

**Affiliations:** 1 Department of Neuroradiology, University Hospital Bonn, Venusberg-Campus 1, 53127 Bonn, Germany; 2 Department of Neurosurgery, University Hospital Bonn, Venusberg-Campus 1, 53127 Bonn, Germany; 3 Department of Neurology, University Hospital Bonn, Venusberg-Campus 1, 53127 Bonn, Germany; 4 Department of Radiology, University Hospital Bonn, Venusberg-Campus 1, 53127 Bonn, Germany

**Keywords:** Magnetic resonance imaging, Essential tremor, Parkinson disease, High-intensity focused ultrasound ablation

## Abstract

**Purpose:**

Magnetic resonance-guided focused ultrasound (MRgFUS) systems are increasingly used to non-invasively treat tremor; consensus on imaging follow-up is poor in these patients. This study aims to elucidate how MRgFUS lesions evolve for a radiological readership with regard to clinical outcome.

**Methods:**

MRgFUS-induced lesions and oedema were retrospectively evaluated based on DWI, SWI, T2-weighted and T1-weighted 3-T MRI data acquired 30 min and 3, 30 and 180 days after MRgFUS (*n* = 9 essential tremor, *n* = 1 Parkinson’s patients). Lesions were assessed volumetrically, visually and by ADC measurements and compared with clinical effects using non-parametric testing.

**Results:**

Thirty minutes after treatment, all lesions could be identified on T2-weighted images. Immediate oedema was rare (*n* = 1). Lesion volume as well as oedema reached a maximum on day 3 with a mean lesion size of 0.4 ± 0.2 cm^3^ and an oedema volume 3.7 ± 1.2 times the lesion volume. On day 3, a distinct diffusion-restricted rim was noted that corresponded well with SWI. Lesion shrinkage after day 3 was observed in all sequences. Lesions were no longer detectable on DWI in *n* = 7/10, on T2-weighted images in *n* = 4/10 and on T1-weighted images in *n* = 4/10 on day 180. No infarcts or haemorrhage were observed. There was no correlation between lesion size and initial motor skill improvement (*p* = 0.99). Tremor reduction dynamics correlated strongly with lesion shrinkage between days 3 and 180 (*p* = 0.01, *R* = 0.76).

**Conclusion:**

In conclusion, cerebral MRgFUS lesions variably shrink over months. SWI is the sequence of choice to identify lesions after 6 months. Lesion volume is arguably associated with intermediate-term outcome.

## Introduction

Magnetic resonance-guided focused ultrasound (MRgFUS) for incisionless cerebral lesional therapy in tremor patients is increasingly gaining interest.

The MRgFUS system delivers energy via sonic elements to a deep brain location in order to thermally create a millimetre-sized strategic lesion. In tremor patients, the target location is currently most often the ventral intermediate nucleus (Vim) of the thalamus. MRI allows for lesion positioning/localisation, as well as temperature control during the procedure. The functional effects of the procedure can clinically be evaluated before a permanent cavity is created; thus in case of unwanted side effects, the lesion position can still be modified. To create a permanent lesion, local temperatures above 54 °C are maintained over several seconds. However, techniques may vary, and lesions may also be achieved at lower temperatures and different energy application techniques, which influence lesion sizes [1–3].

Although MRgFUS is a more recent method to treat movement disorders, several thousand patients have already been treated worldwide. Radiologists therefore may be confronted with patients after MRgFUS therapy, and thus should have knowledge on lesion development after the procedure [2, 4–11].

Based on an extended image analysis from the day of treatment until 6 months post-intervention, this study evaluated thalamic lesions after MRgFUS in ET and PD patients. Particular emphasis was put on the suspected position of Vim versus actual position of the lesion, and also the clinical presentation after intervention.

## Methods

### Summary of the MRgFUS procedure

**Fig. 1:**
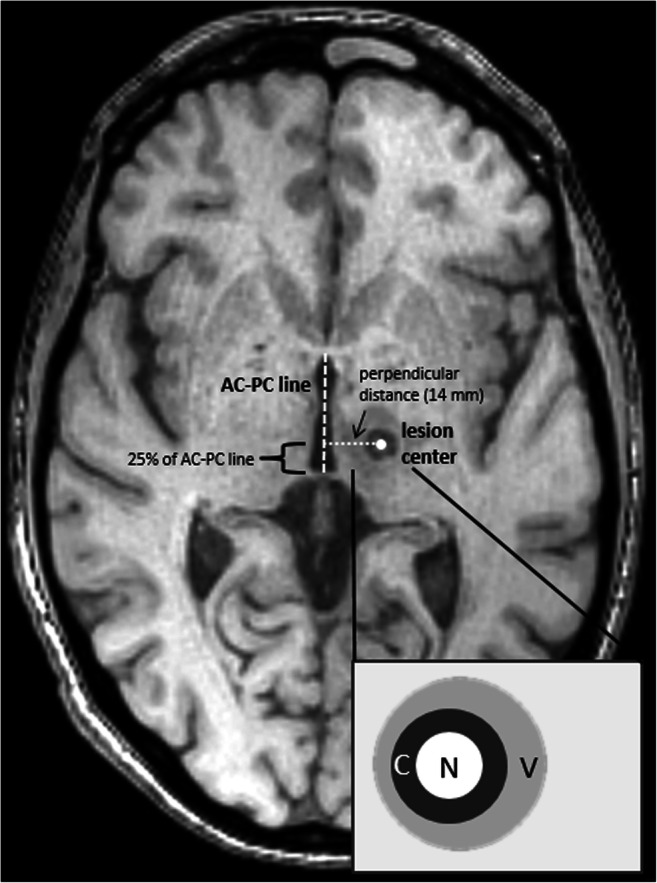
T1-weighted Vim lesion image on day 3. The left-hemispherical ventral intermediate nucleus (Vim) lesion was expected about 14 mm left and centrally 1 mm on the anterior-posterior commissure line (AC-PC line) approximately 25% of the total distance ventral of the PC. The enlarged scheme illustrates the expected necrotic core (N), the ring-shaped cytotoxic oedema zone (C) and the blurred vasogenic oedema (V)

The MRgFUS procedure at our institution is performed with the Exablate Neuro 4000 (InSightec, Haifa, Israel) integrated into a 3-T MRI system (GE Discovery 750, GE Healthcare, Milwaukee, WI, USA). Patients are awake during the so-called sonications (ultrasound applications), which allows a real-time evaluation of the clinical ultrasound effects. In all patients, the thalamic Vim was unilaterally treated. If the treatment effect is based on targeting the Vim itself or nearby fibre tract structures is at present subject of debate [8]. In accordance with the literature, the Vim centre was defined to be on the level of the anterior-posterior commissure (AC-PC) line and 14 mm lateral to it. The anterior-posterior position was one quarter of the total AC-PC line distance anterior of the PC (Fig. 1) [12]. This target however is only intended as a starting point for trial sonications of lower energy leaving no permanent lesion. Lesions were eventually produced based on the spot of the test sonication that showed the best clinical improvement. The number of (test) sonications, the necessity of energy delivered to achieve a certain temperature and the peak temperature itself are highly individual, but are protocoled for case management. A typical sonication lasts 14 to 20 s depending on the energy that needs to be delivered.

### Patients

Ten patients were included in this retrospective observational study: nine with essential tremor (ET) and one with tremor-dominant Parkinson’s disease (PD). Six were men and four women with a mean age 69.7 ± 8.6 years. All had undergone MRgFUS treatment of the Vim (left/right Vim 9/1 patients respectively) with intrainterventional 3-T MRI, and repetitive 3-T MRI as part of the post-interventional monitoring plan.

After written-informed consent, patients had enrolled for MRgFUS treatment and follow-up based on clinical inclusion criteria defined as part of this German Clinical Trials registered and ethically approved study (DRKS00016695). This imaging study follows STROBE guidelines for observational studies.

### Imaging follow-up

The first MRI was performed within 30 min of the treatment (day 0), while patients were still in the 3-T treatment unit (GE Discovery MR750w, Chicago, IL, USA), using the integrated body coil. The three follow-up MRIs (3 days, 1 month, and 6 months after treatment, termed days 3, 30, 180) were performed with an 8-channel head coil using a different 3-T MRI system (Philips Achieva, TX).

The patients still received dexamethasone (4 mg oral, thrice daily from day 0 until day 5) during the day 3 MRI.

### 3-T MRI protocol

The control scan immediately after the intervention consisted of an axial T2-weighted sequence only. In the further follow-up, scans involved a 3D T1-weighted MPRAGE sequence, an axial T2-weighted sequence, a diffusion weighted (DWI), and a susceptibility-weighted (SWI) sequence (Table 1). The SWI image consisted of a phase and a magnitude image of which the magnitude image was used for analysis. Detailed sequence information is given in Table 2.

**Table 1:** Temporal scheme of MRI scans after MRgFUS

Sequence	Day 0	Day 3	Day 30	Day 180
T2w	x	x	x	x
SWI		x	x	x
DWI		x	x	x
T1w		x	x	x

*DWI* diffusion-weighted imaging, *SWI* susceptibility-weighted imaging, *T1w* T1-weighted, *T2w* T2-weighted

**Table 2:** Sequence parameters

Sequence	Pulse type	Orientation	TR (ms)	TE (ms)	Reconstructed voxel size (mm)	Matrix (mm)	Slices	Gap (mm)	Scan time
Day 0: T2w	T2 propeller	Axial	10,991	113	0.45 × 0.45 × 2	512 × 512	30	0.5	5′ 19″
T2w	Turbo spin echo	Axial	13,257	90	0.94 × 0.94 × 1	240 × 174	140	0	5′ 45″
SWI	3D fast field echo	Axial	31	0	0.6 × 0.6 × 2	384 × 316	145	0	3′ 57″
DWI	*b* values (0, 500, 1000 s/mm^2^)	Axial	2725	41	1 × 1 × 5	128 × 127	24	1	0′ 49″
T1w	MPRAGE	Sagittal 3D	7.3	3.9	1 × 1 × 1	256 × 256	180	0	4′ 39″

The total protocol length for follow-up (days 3 to 180) remains below 15 min

*DWI* diffusion-weighted imaging, *MPRAGE* magnetization prepared-rapid gradient echo, *SWI* susceptibility-weighted imaging, *T1w* T1-weighted, *T2w* T2-weighted, *TE* echo time, *TR* repetition time

### Image analysis

Two neuroradiologists (9 and 6 years of experience) performed blinded intensity threshold-based ROI volumetry and additional two-dimensional diameter measurements in all lesions based on T1-weighted, T2-weighted and SWI sequences at all applicable time points on separate work stations (Intellispace 8.0, Philips Healthcare). The volume borders included the presumed cytotoxic oedema zone assuming unsalvageable tissue in this area forming the later cavity.

Deviation between the actual lesion position and the position suggested in the literature was assessed on T1-weighted images on day 3 scans [12]. As already mentioned, the planned Vim centre was defined to be on the level of the AC-PC line and 14 mm lateral to it. The suggested anterior-posterior lesion position was one quarter of the total AC-PC line distance anterior of the PC (Fig. 1) [12]. For measurements, the lesion centre was considered to be perpendicular to the AC-PC line.

Presence and volume of perilesional oedema were assessed on T2-weighted images.

ROIs were placed in the image slice depicting the largest area of the lesion to measure dynamics of the ADC values over time.

T1-weighted images were visually assessed for the presence of lesions corresponding to lesions with low signal on SWI as an indicator of desoxyhemoglobin or methemoglobin that can be expected from day 3 on [13].

### Clinical evaluation

Patients were evaluated by two neurologists (28 and 5 years of experience) before MRgFUS and on all days of MRI follow-up. Clinical symptoms were registered in detail. For comparison with imaging data, however, two classification sets were applied translated from the patients’ scores either on the Unified Parkinson’s Disease Rating Scale (UPDRS) in PD or the Fahn-Tolosa-Marin Tremor Rating Scale (TRS) in ET:

1. Clinical rating:

1 = tremor symptoms at least 75% reduced, 2 = tremor at least 50% reduced compared to baseline, 3 = tremor maximally reduced by 50%, 4 = tremor as strong as before MRgFUS or worse.

2. Side effects:

0 = none, 1 = dysaesthesias, 2 = gait instability, 3 = dysarthria, 4 = paresis.

### Statistical analysis

Statistics were performed in SPSS 24.0 using non-parametric testing including paired Wilcoxon tests for inter-time point and sequence comparisons and Spearman rank correlation for clinical outcome versus lesion volume (IBM Corp.). Volume dynamics were compared in absolute values and ratios to a baseline value (defined as 100% and describing the lesion during its largest volume on average), while all other measurements were compared in absolute values only. Intraclass correlation (two-way mixed effect model) between both neuroradiological readers was determined. Reliability of measurements were considered as poor with an intraclass correlation coefficient (ICC) below 0.5, as moderate between 0.5 and 0.75, as good between 0.75 and 0.9 and as excellent above 0.9 [14].

## Results

### Lesion location

Mean AC-PC line length was 25.5 ± 2.0 mm (range 22.0–29.6 mm). Clinically determined centres of Vim lesion placement differed from the assumed position of the nucleus as suggested in the literature.

On the right-left axis, mean lesion position was 0.5 ± 1.3 mm (mean and standard deviation) lateral to the expected 14 mm (range 1.3 mm more medial to 3.2 mm more lateral), and 2.9% more anterior than the expected 25% distance from the posterior commissure on the AC-PC line (range − 2.3 to 6.9%; Table 3). Minimum distance between the AC-PC line and the centre of the lesion was 12.7 mm; the maximum distance was 15.7 mm. The lesion centre on the cranio-caudal axis was on average 1.1 ± 1.2 mm above the AC-PC line (range 1 mm below to 3 mm above; Table 3).

**Table 3:** Lesion location and clinical outcome

Patient	Deviation (cc in mm/rl in mm/ap in %)	Before treatment	Day 3				Day 30			Day 180			
		Clinical symptoms	T2 lesion volume (cm^3^)	Tremor control treated side	Side effects	Clinical symptoms	T2 lesion volume (cm^3^)	Tremor control treated side	Side effects	T2 Lesion volume (cm^3^)	Tremor control treated side	Side effects	Clinical symptoms
P1	0/0/1.1	• Minor head tremor • Minor rest tremor of right arm • Marked postural and intention tremor of arms	0.3	1	3	• Head tremor • And rest tremor of right arm gone • Minor intention tremor right arm • Left side unchanged • **Subjective dysarthria**	–	2	0	0	1	0	• Stable clinical effect • **AE fully resolved**
**P2**	3/0.2/4.3	• Minor head tremor • Moderate voice tremor • Severe postural and intention tremor of arms • Minor postural and intention tremor of legs	0.5	1	1	• Minor head tremor • Minor voice tremor • Minor intention tremor right-sided extremities • Left side unchanged • **Unsteady feeling, tingling of fingertips and perioral region**	0.1	2	1,2	0.1	4	1	• *Moderate head tremor* • *Moderate voice tremor* • *Moderate intention tremor right arm* • *Severe intention tremor left arm* • **AE constant**
**P3**	1/3.2/6.4	• Moderate head tremor • Marked postural and intention tremor of arms	0.8	1	4	• Minor head tremor • Minor intention tremor of right arm • Moderate postural and intention tremor *left* arm • **Moderate hemiparesis of right leg > arm, dysarthria, facial nerve palsy right**	0.5	2	1,3,4	0.1	3	3,4	• *Gradually fading clinical effect* • **Residual minor paresis of right leg**
P4	2/1.7/4.3	• Moderate head tremor • Minor voice tremor • Minor rest tremor of arms • Severe postural tremor of arms • Moderate intention tremor of arms • Minor postural and intention tremor of legs	0.2	1	0	• Minor head and voice tremor • Minor postural and intention tremor left arm • No tremor of left leg • Right side unchanged • **No AE**	0.1	2	0	0	1	0	• Mild deterioration in head tremor, otherwise stable • **No AE**
P5	− 1/− 1/6.4	• Moderate head tremor • Minor rest tremor of arms and postural tremor of legs • Marked postural and intention tremor arms	0.4	1	0	• Minor intention tremor right arm • Moderate tremor left arm and leg • **No AE**	0.1	1	0	0	1	0	• Right side stable • Left side like before treatment • **No AE**
P6	0/− 0.3/6.9	• Minor head and postural/ intention tremor of legs • Marked postural and intention of arms	0.2	1	0	• No more head tremor, no more tremor right extremities • Minor improvement in left extremity tremor • **No AE**	0.1	1	2	0	1	0	• Clinically stable • **Subjective unsteady feeling not verified by clinical tests and probably not due to treatment**
P7	2/− 1.3/− 1.7	• Marked postural and intention tremor of arms	0.4	1	1	• Minor intention tremor right arm • Moderate tremor in left arm • **Tingling lips right side**	0.2	1	1	0	1	1	• Clinically stable • **AE resolved**
P8	0/0.3/− 2.3	• Minor head and voice, tremor minor postural and intention tremor of legs • Marked postural tremor of arms • Moderate intention of arms	0.2	1	2	• Minor intention tremor right arm • Other extremities like before treatment unchanged • **Unsteady feeling while standing**	0.1	1	1	0	1	1,2	• Clinically stable • **AE slightly better**
P9	2/0.5/− 1.6	• Minor head and voice tremor minor postural and intention tremor of legs • Severe postural/intention tremor of arms	0.5	2	0	• Minor improvement in voice, right leg and left arm tremor • Minor intention tremor right arm • **Paresthesia fingers right hand**	0.2	2	2	0.1	2	1	• Clinically stable • **AE partially resolved**
P10	2/1.2/5.5	• Moderate rest tremor of right arm • Marked postural tremor right>left arm • Severe intention tremor right>left arm • Moderate bradykinesia and rigour of right arm, updrs iii =17	0.6	1	0	• No tremor of right arm • Moderate improvement of left arm • **No AE**	0	1	0	0	1	0	• Clinically stable • **No AE**

Deviation: distance of actual lesion centre from assumed centre of Vim. Minus on cc axis equals more caudal than anterior-posterior commissure (AC-PC) level; minus on rl axis equals more medial than 14 mm lateral to AC-PC line; minus on ap axis equals less than 25% of total AC-PC line distance away from PC. Number encoding for tremor control: 1 = tremor symptoms at least 75% reduced, 2 = tremor at least 50% reduced compared to baseline, 3 = tremor maximally reduced by 50% and 4 = tremor as strong as before MRgFUS or worse. Number encoding for side effects*:* 0 = none, 1 = dysaesthesias, 2 = gait instability, 3 = dysarthria and 4 = paresis. Patients with secondary deterioration/reduction of positive effect marked in bold with symptoms marked in italic (P2 and 3)

*AE* adverse effect in bold, *ap* anterior-posterior, *cc* cranio-caudal, *rl* right-left, *UPDRS* Unified Parkinson’s Disease Rating Scale

### Lesion signal intensity and dimensions

**Fig. 2:**
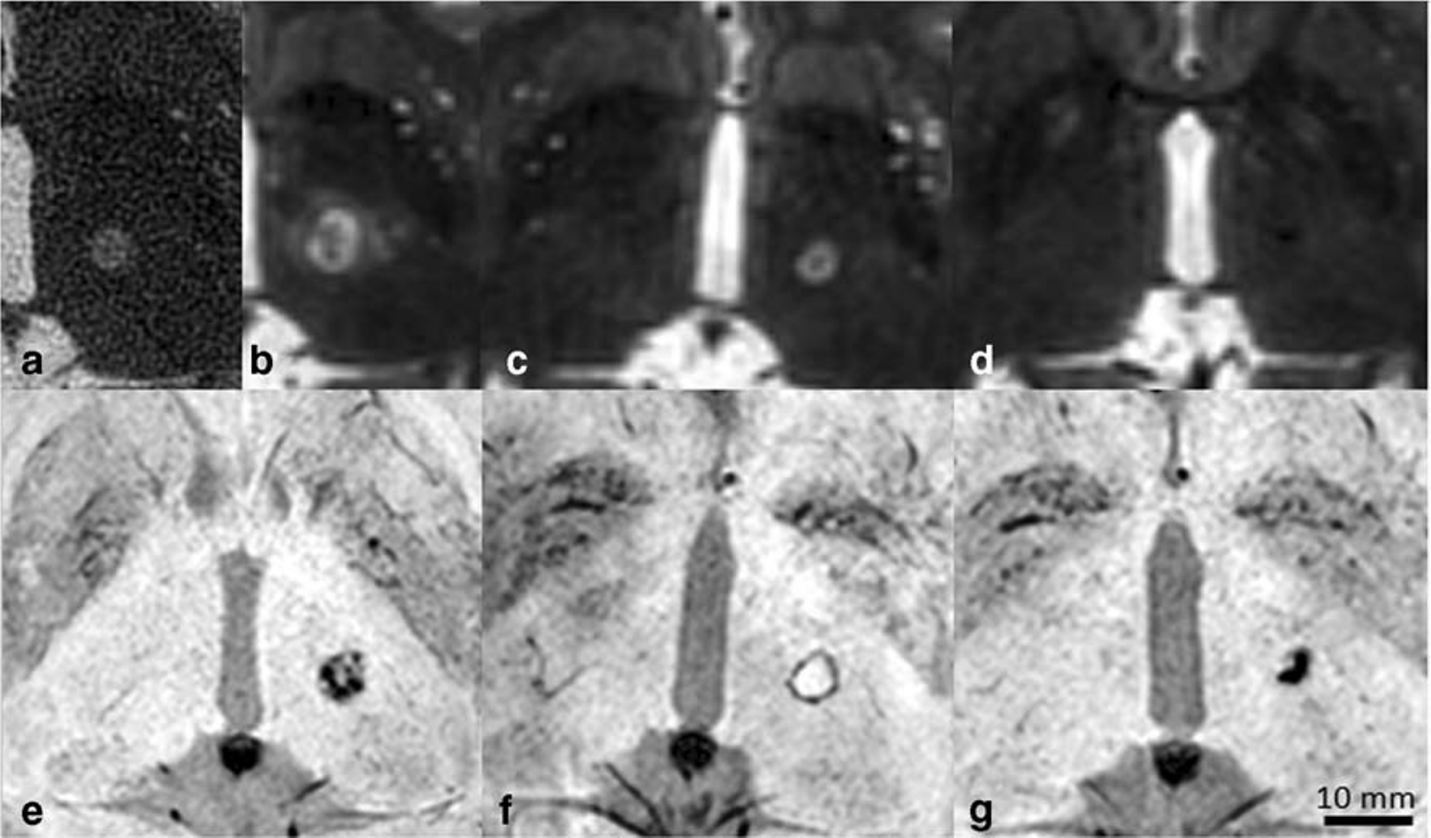
Lesion dynamics at 3-T on T2-weighted and susceptibility-weighted images over time. **a**–**d** T2-weighted images and **e**–**g** susceptibility-weighted images. **a** Thirty minutes after therapy, **b** 3 days, **c** 30 days and **d** 180 days after therapy. **e** Three days, **f** 30 days and **g** 180 days after therapy. Note how the lesion first increases in size during the first 3 days (**a** vs. **b**), while it has completely vanished half a year after therapy on T2-weighted images and yet remains distinctly visible on susceptibility-weighted images. Image b also depicts the oedema surrounding the core lesion separated by a fine hypointense dark rim. The noisy aspect of image a is due to the distant MRI-integrated body coil used, while the patient was still wearing the treatment helmet

**Fig. 3:**
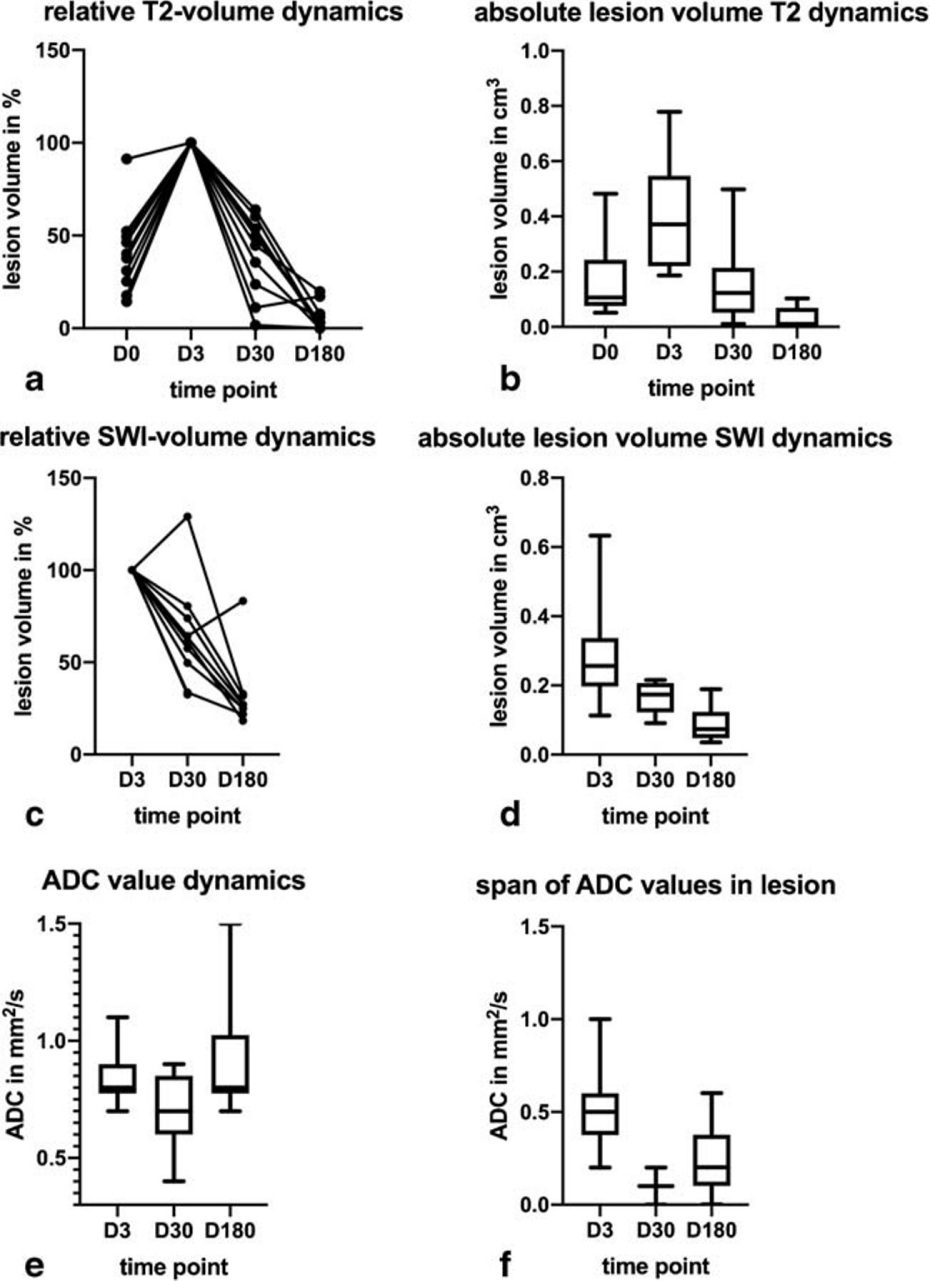
Temporal evolution of ventral intermediate nucleus lesion volumes and corresponding apparent diffusion coefficients (ADCs). Dimensionless relative values are defined as relative to the volume measured on day 3 by building the ration (*x*_*t*_ / *x*_*d*_ = 3). **a** Relative lesion volume dynamics measured on T2-weighted images in 10 patients. **b** Absolute lesion volumes on T2-weighted images marked by mean and 5th to 95th percentiles. **c** Relative lesion volume dynamics measured on susceptibility-weighted images. **d** Absolute lesion volumes on susceptibility images marked by mean and 5^th^ to 95^th^ percentiles. **e** Absolute ADC values at different time points stated as mean and 5^th^ to 95^th^ percentiles. **f** The span of ADC values describes the difference between the minimum and maximum ADC measured in a lesion region of interest at a point of time. D0: day 0 MRI 30 min after therapy, D3: MRI on day 3 after therapy, D30: MRI 30 days after therapy, D180: MRI 180 days after therapy

Lesion signal intensities differed per time point and sequence (Table 4). Lesion volumes and diameters were temporally dynamic with significant absolute and relative differences between sequences at all time points (Fig. 2). Mean T2-weighted imaging lesion volumes on day 0 were only 42.5 ± 20.5% of the volume they reached on day 3 (Figs. 2 and 3).

**Table 4:** Temporal signal intensity evolution of lesions after MRgFUS in comparison with surrounding thalamus

Sequence	Day 0	Day 3	Day 30	Day 180
T2w	Rim: hyper Centre: hypo	All hyper	All hyper	All hyper
SWI	n/a	Rim: hypo Centre: hyper	Rim: hypo Centre: hyper	All hypo
DWI	n/a	Rim: low ADC Centre: high ADC	All high ADC	All intermediate or slightly elevated ADC
T1w	n/a	All hypo	All mildly hypo	All mildly hypo to iso

*ADC* apparent diffusion coefficient, *DWI* diffusion-weighted imaging, *SWI* susceptibility-weighted imaging, *T1w* T1-weighted, *T2w* T2-weighted, *centre* lesion centre, *hyper* hyperintense in comparison with healthy thalamus, *hypo* hypointense in comparison with healthy thalamus, *iso* isointense in comparison with healthy thalamus, *rim* outer lesion rim

On day 3, the day of largest lesion volume, mean lesion volume (T2-weighted images) was 0.4 ± 0.2 cm^3^ (minimum 0.2 cm^3^, maximum 0.8 cm^3^) and 0.3 ± 0.1 cm^3^ (minimum 0.1 cm^3^, maximum 0.7 cm^3^) on SWI images. This volume difference was significant (*p* = 0.007). However, after day 3, volumes and diameters shrank until end of observation on day 180 with mean SWI-measured lesion volumes being larger than those measured on T2-weighted images (Figs. 2 and 3). Indeed, in *n* = 4/10 cases, lesions were no longer discernable on T2-weighted images, while the SWI images still showed a clear lesion in all cases.

On day 180, lesion volumes had shrunk to 6.0 ± 6.7% of the peak volume measured on day 3 for T2-based volume measurements (*p* = 0.005) and to 44.1 ± 42.4% of the day 3 peak volume on SWI (*p* = 0.008), leaving a significant difference in volumes also between both sequences performed on day 180 (*p* = 0.008).

**Fig. 4:**
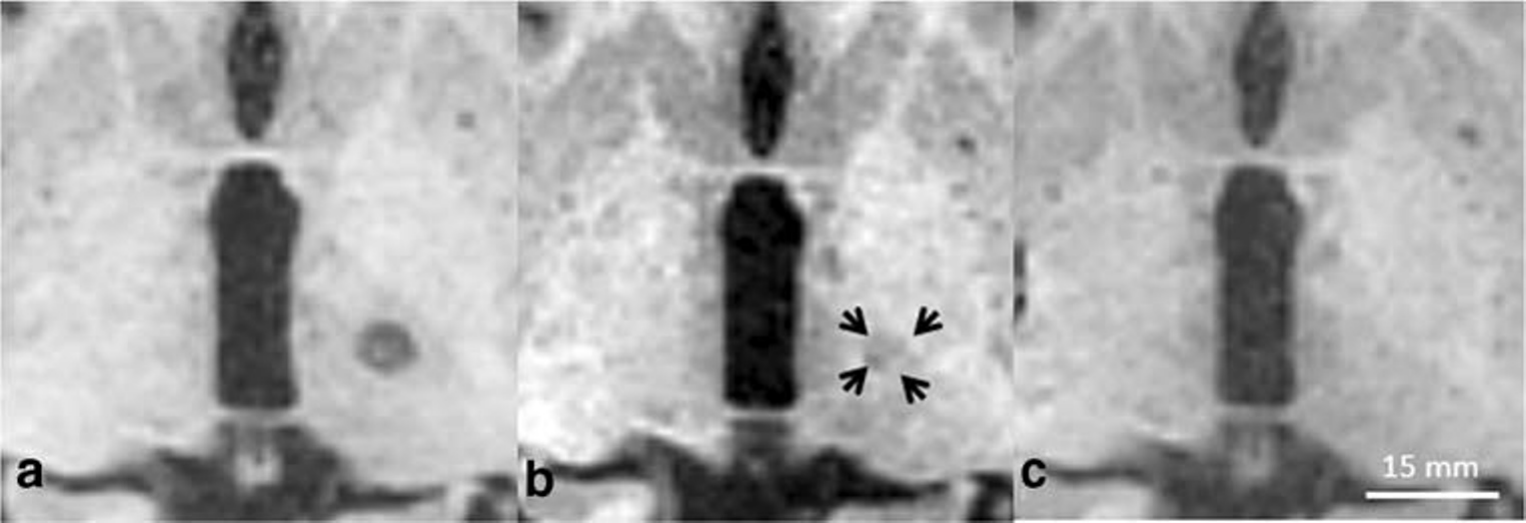
Fading of the MRgFUS lesion on T1-weighted images. While on SWI and T2-weighted images shrinkage of the lesion was noted, the lesion rather faded in signal intensity compared with the surrounding brain tissue. **a** Day 3, **b** day 30 (black arrows indicating lesion margins) and **c** “vanished” lesion on day 180

On T1-weighted images, the mean relative volume on day 180 was 8.2 ± 10.2% of the volume of day 3 (range 0.0 to 31.6%). Additionally, a fading of the lesion was noted with decreasing hypointensity of the lesion in contrast to the adjacent deep grey matter (Table 4**,** Fig. 4). In consequence, lesions were no longer discernable in *n* = 4/10 cases on T1-weighted images on day 180.

Lesion diameters were comparable across T1-weighted, T2-weighted and SWI sequences (*p* = 0.78), and all reached their maximum volume on day 3. All were below 10 mm in the long axis and 8 mm in the short axis except for one patient with a lesion measuring 16.4 × 10.5 mm on T2-weighted images (mean 8.4 ± 2.2 mm long axis, mean 6.4 ± 1.6 mm short axis), who showed clinical side effects (see below). The initial mean diameters 30 min after therapy were 6.1 ± 1.9 mm (long axis) and 4.6 ± 1.4 mm (short axis), and thus more than 20% shorter in each direction (all measurements on T2-weighted images).

#### Oedema formation and diffusivity

Immediately after MRgFUS therapy, mild oedema formation was observed in only one patient (0.1 cm^3^). The day 3 control MRI showed oedema in *n* = 9/10 cases (mean volume 1.5 ± 0.9 cm^3^, minimum 0 cm^3^, maximum 4.0 cm^3^; Fig. 2b). This fully resolved by day 30.

Mean oedema volumes measured 3.7 ± 1.2 times the lesion itself on day 3 (measured on T2-weighted images). Oedema volume correlated well with lesion size on SWI and T2-weighted volumetry (*R* = 0.824, *p* = 0.003 and *R* = 0.707, *p* = 0.022 respectively).

In only 4/9 cases, vasogenic oedema extended beyond the thalamus showed a predilection to extend to the white matter tracts of the internal capsule.

At no point in time, patients showed changes of diffusivity suspicious of infarcts outside the MRgFUS lesion.

**Fig. 5:**
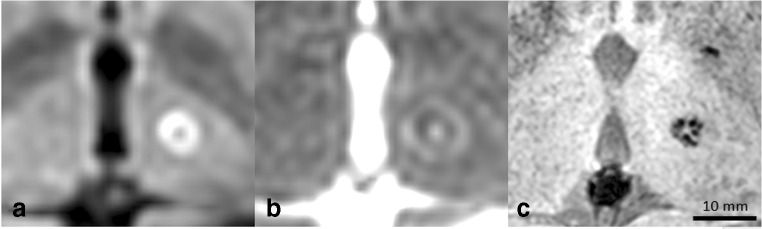
Lesion presentation on diffusion-weighted images on day 3 after therapy and corresponding susceptibility-weighted image. **a***b* = 1000 s/mm^2^ diffusion-weighted image depicting a characteristic bright ring formation inside the lesion. **b** According to the low intensity on the apparent diffusion coefficient map, the lesion has restricted diffusivity. **c** The ring shape corresponds well to the speckled ring shape of the lesion observed on susceptibility-weighted images of the same person

Diffusivity dynamics between days 3 and 180 were more complex than volume dynamics (Fig. 3). Between days 3 and 30, mean ADC dropped (0.8 ± 0.1 to 0.7 ± 0.2 mm^2^/s, *p* = 0.07), rising again between days 30 and 180 to 0.9 ± 0.3 mm^2^/s (*p* = 0.04). The early lesions on day 3 all showed a peculiar ring formation of lower ADC with a centrally higher ADC that corresponded well with the ring formation observed on SWI (Fig. 5). On days 30 and 180, the lesion was either uniform (*n* = 9/10 on day 30; *n* = 3/10 on day 180) or no longer discernable as a lesion on ADC maps at all (*n* = 1/10 on day 30; *n* = 7/10 on day 180). The span of ADC values measured inside the lesion hence was larger on day 3 due to differences measured in the ring formation than on day 30 (Fig. 3).

### Technical data and clinical correlation

**Fig. 6:**
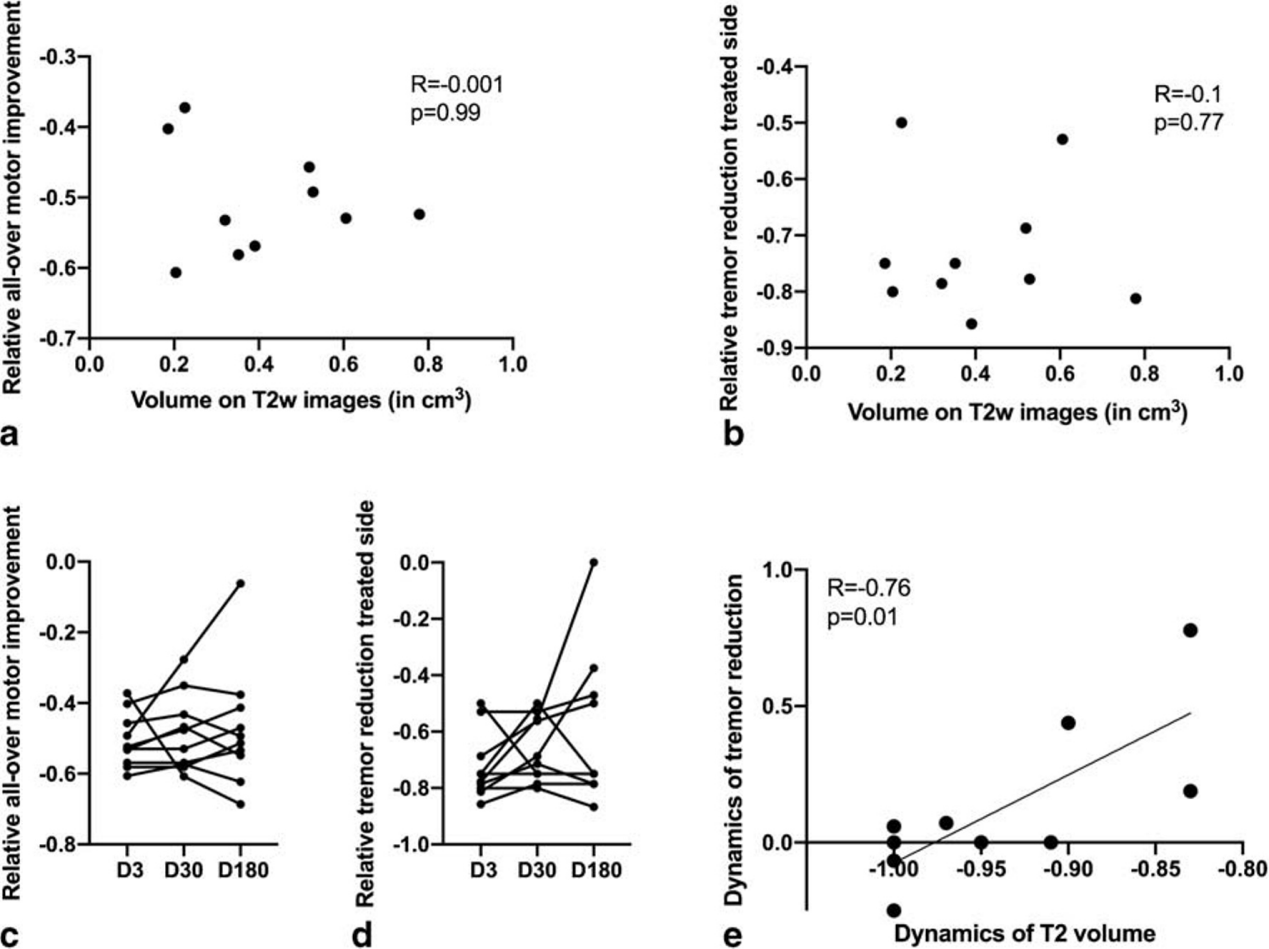
**a** Reduction of motor symptoms in general motor task testing as opposed to baseline (baseline score minus post-treatment score/baseline score) correlated with lesion volume both on day 3. **b** Reduction of tremor symptoms on the treated side as opposed to baseline (baseline score minus post-treatment score/baseline score) correlated with lesion volume both on day 3. **c** Relative reduction of motor symptoms in general motor task testing over time. **d** Relative reduction of tremor symptoms on the treated side over time. **e** Dynamics of relative changes in tremor reduction (day 180 versus day 3) as opposed to dynamics in T2 volume between day 180 and day 3. *A* − 1.00 on the *x*-axis represents a lesion that is no longer discernable. D3: MRI on day 3 after therapy, D30: MRI 30 days after therapy, D180: MRI 180 days after therapy, T2w: T2-weighted volumetry

Table 5 illustrates the technical parameters to achieve a lesion for each of the patients. There was no significant correlation between initial lesion volume and clinical improvement (*p* = 0.99 for all-over motor improvement and *p* = 0.77 for tremor reduction on the treated side; Table 3; Fig. 6a, b).

**Table 5:** Individual patient treatment course

Patient	Diagnosis	Number of test sonications	Number of therapeutic sonications	Highest achieved temperature (°C)	Energy delivered (W)
1	ET	7	4	62	850
2	ET	10	5	61	756
3	ET	8	9	60	846
4	ET	9	5	58	894
5	ET	11	9	61	900
6	ET	5	5	64	849
7	ET	7	4	65	929
8	ET	7	4	61	1099
9	ET	9	4	62	1001
10	PD	4	3	59	900

The energy applied represents the energy truly delivered into the tissue, not the a priori determined value

*ET* essential tremor, *PD* Parkinson disease

Clinical effects remained stable in the majority of patients during the first 180 days (*p* = 0.43 all-over motor skills; *p* = 0.29), despite rare secondary deterioration of all-over motor skills (*n* = 2) or tremor on the treated side (*n* = 2; Fig. 6c, d).

Greater lesion shrinkage was not correlated with all-over motor improvement (*p* = 0.57), but with dynamics of tremor improvement of the treated side clinical outcome (*p* = 0.01, *R* = 0.76; Fig. 6e). One patient (patient 3), whose lesion was particularly far lateral from the AC-PC line (17.2 mm) and showed extensive oedema (day 3 maximum 4.0 cm^3^), developed moderate hemiparesis with not onlt secondary improvement regaining walking capacity, but also secondary deterioration of the therapeutic effect (Table 3).

### Interobserver reliability

Interobserver reliability lesion measurements were excellent for T2-weighted (ICC 0.99 confidence interval [0.99, 0.99]), T1-weighted (ICC 0.99 [0.99, 0.99]) as well as for oedema volumes (ICC 0.92 [0.90, 0.94]) and good for SWI (ICC 0.89 [0.75, 0.95]). Reliability of ADC measurements was excellent (ICC 0.96 [0.92, 0.98]). Lesion diameter measurements were also reliable for T2-weighted, SWI and T1-weighted with ICCs of 0.99.

#### Discussion

This study elaborated the evolution of imaging features of MRgFUS thalamic lesions. MRgFUS lesions shrink within the first 6 months after therapy. Lesions may become invisible on DWI, T1- and T2-weighted images, while as a main finding, they always remained identifiable on SWI. DWI is excellent to reveal the borders of the cytotoxic oedema right after therapy, but resolves within 1 month. Our data further suggests that while there seems to be no association between lesion volume and clinical improvement in the acute phase, there are signs that dynamics of lesion shrinkage can be associated with dwindling tremor improvement as is suggested by better tremor improvement of the treated side being correlated to less lesion volume reduction.

The initial enlargement of the MRgFUS lesions between days 0 and 1 with secondary shrinkage of lesion volume on T2-weighted images is a common feature [15, 16]. Our findings suggest that T2-weighted images can be inadequate to assess MRgFUS already after 1 month due to complete disappearance of the lesion. As T2-weighted images may return to normal after 1 month, these seem inadequate for longer follow-up studies. However, they are primarily useful in the first days after MRgFUS therapy to assess oedema formation and approximate lesion extent.

It is not surprising that lesion volumes differ significantly between T1- and T2-weighted studies [3], and also when comparing SWI and T2-weighted images as in this study, as the ultrasound effect on the lesion is not uniform creating variable tissue changes that result in likewise variable alteration in T1 and T2 signal. SWI is mainly used to identify cerebral haemorrhage and discriminate it from calcification, and we do know from clinical experience that SWI has the technical capacity to detect very subtle hemosiderin remnants where T1 and T2-weighted sequences of a similar resolution do not show any traces [17]. The outstanding lesion persistence of low signal on SWI compared with other sequences and also T2* GRE is well documented for post-traumatic as well as subarachnoid haemorrhage lesions [18, 19]. All lesions in our study showed a *low* signal on SWI representing coagulation necrosis/infarct-like thermal tissue alterations as demonstrated histologically by Elias et al. in a porcine model [20]. The most likely explanation is the focal destruction of erythrocytes in the MRgFUS lesion centre. On the other hand, due to these low signal lesions, it can be problematic to identify true intralesional bleeds deriving from vessel damage within this zone with SWI as they would appear the same and are therefore camouflaged. Haemorrhage after cerebral MRgFUS is however rare (*n* = 0/10 in this study). Yet, single reports of microbleeds exist with haemorrhage seen as bright spots from methemoglobin after day 3 on T1-weighted images, which can be used for bleed verification [21].

We consistently observed a fading of decreasingly hypointense lesions on T1-weighted images on day 30. Wintermark et al. disparately infrequently identified bright ring lesions on this day [4]. The temporal dynamics towards an eventual fading of lesions on T1-weighted images is however identical. This fading on T1-weighted images may be explained by biochemical changes, e.g. protein degradation and iron-containing particle removal, in the composition of the lesion over time, which shortens T1 relaxation time. The phenomenon of temporal alteration in T1 signal was recently published in a longitudinal case report on radiation necrosis describing significant alterations in T1 relaxation time and SWI aspect over a period of 52 months [22]. However, it does not explain the difference in intensity on day 30 between our cohorts and in part that of Wintermark. The radiologist must bear mind that effects other than T1 shortening and secondary fading may be observed.

The imaging aspect of MRgFUS lesions seen on SWI over time suggests a condensation of necrotic tissue elements from a ring-like shape (or spherical in 3D) to a contracted granular end-stage lesion, which shows parallels to treatment of the VIM using radiofrequency probes in the past [23]. DWI may indirectly confirm this as according to our study, a ring-shaped diffusion-restricted structure representing cytotoxic oedema matched excellently with the ring structure observed in SWI on day 3. Again, similar to T2-weighted imaging, DWI was only useful during the first days after MRgFUS to show the intralesional cytotoxic oedema.

Vasogenic oedema was also a short-term phenomenon and—with one exception—not present before day 3 and no more observed beyond day 30. Although only present in one case in our study, there was a predilection of oedema to follow white matter structures along the internal capsule and hypothalamic tracts with relative sparing of deep grey matter structures, which is anatomically plausible due to water following the anisotropic lead structures set by white matter bundles.

Clinically based lesion positions differed slightly from the assumed position of the Vim in our study, an observation that had been reported earlier based on DTI data [24]. All-over analysis in this study showed a non-significant reduction of treatment effects until 6 months after MRgFUS. However, lesions with larger relative reduction in volume within 6 months correlated with a more prominent recurrence of tremor. It can be speculated that in these patients, at least some of the initial effect was due to lesional oedema. These two observations are in coherence with a 2-year follow-up study in ET patients, that similar to our study did not identify any delayed effects [11], but partially stands against findings of Wintermark et al., who showed a clear decline of clinical effect with declining oedema and lesion size [4]. Even in retrospect, it remains unclear why two of our patients showed a measurable deterioration of symptom control, i.e. secondary treatment failure. There was no difference in distance between real and expected target or lesion volume decline in those two compared with the others. Lesion size and presence of oedema were indeed remarkably larger in patient 3, who also had substantial side effects. However, this is not true for patient 2 and does also not explain a secondary reduction of effect. Another aspect can be the technical performance of the treatment. Still no differences regarding the number of test sonications, peak temperatures or energy applied can be identified in those patients with partial secondary treatment failure. These technical parameters are however anyhow highly individual for the patient and the lesion and depend on multiple factors such as the skull density score, previous sonications and temperature evolution. Highly interesting is also the positive therapeutic effect to the non-treated side after unilateral MRgFUS, which in some cases was permanent in our patients. The underlying reason is most likely an effect on crossing fibres to the contralateral hemisphere. The phenomenon was previously described for MRgFUS and deep brain stimulation alike, but still needs further scientific exploration, e.g. with fibre tracking [25].

While our study is based upon a very limited number of patients, its results are in line with previous findings. The diversity of MRgFUS techniques remains however an unknown influential factor in this context.

In conclusion, radiologists should be aware that the follow-up of MRgFUS lesions shows an ageing and especially shrinkage process and therefore require different high-resolution sequences for follow-up. Lesions can be observed longer on SWI than on other sequences. The clinical effect however outlives the Vim lesion itself, such that it remains questionable, if shrinkage dynamics have any association with the clinical outcome. In consequence, necessity and interpretation of long-term follow-up MRI in MRgFUS patients is disputable.

## Abbreviations

AC-PC: Anterior commissure-posterior commissure
CI: Confidence interval
ET: Essential tremor
ICC: Intraclass coefficient
MPRAGE: Magnetization prepared-rapid gradient echo
MRgFUS: Magnetic resonance-guided focused ultrasound
PD: Parkinson’s disease
Vim: Ventral intermediate (thalamic) nucleus
